# Novel Informatic Tools to Support Functional Annotation of the Durum Wheat Genome

**DOI:** 10.3389/fpls.2019.01244

**Published:** 2019-10-10

**Authors:** Mario Fruzangohar, Elena Kalashyan, Priyanka Kalambettu, Jennifer Ens, Krysta Wiebe, Curtis J. Pozniak, Penny J. Tricker, Ute Baumann

**Affiliations:** ^1^School of Agriculture, Food and Wine, The University of Adelaide, Adelaide, SA, Australia; ^2^Department of Plant Sciences and Crop Development Centre, University of Saskatchewan, Saskatoon, SK, Canada

**Keywords:** exome capture, mutagenesis, reverse genetics, durum wheat, polyploidy, TILLinG

## Abstract

Seed mutagenesis is one strategy to create a population with thousands of useful mutations for the direct selection of desirable traits, to introduce diversity into varietal improvement programs, or to generate a mutant collection to support gene functional analysis. However, phenotyping such large collections, where each individual may carry many mutations, is a bottleneck for downstream analysis. Targeting Induced Local Lesions in Genomes (TILLinG), when coupled with next-generation sequencing allows high-throughput mutation discovery and selection by genotyping. We mutagenized an advanced durum breeding line, UAD0951096_F2:5 and performed short-read (2x125 bp) Illumina sequencing of the exome of 100 lines using an available exome capture platform. To improve variant calling, we generated a consolidated exome reference using the recently available genome sequences of the cultivars Svevo and Kronos to facilitate the alignment of reads from the UAD0951096_F2:5 derived mutants. The resulting exome reference was 484.4 Mbp. We also developed a user-friendly, searchable database and bioinformatic analysis pipeline that allowed us to predict zygosity of the mutations discovered and extracts flanking sequences for rapid marker development. Here, we present these tools with the aim of allowing researchers fast and accurate downstream selection of mutations discovered by TILLinG by sequencing to support functional annotation of the durum wheat genome.

## Introduction

Mutants are valuable tools for the identification and functional analysis of genes. Mutations can arise spontaneously or can be induced physically (e.g., radiation), chemically (e.g., alkylating agents), and by transposon insertions or through gene editing, such as with the CRISPR/Cas9 system for specific modifications of target genes (Adli, 2018).

The use of chemical mutagenesis has had a renaissance with the development of Targeting Induced Local Lesions in Genomes (TILLinG) method in *Arabidopsis* (McCallum et al., 2000). TILLinG is a high-throughput method of inducing and identifying genetic variations in target genes. Its main advantage is that it can be employed as a functional genomics platform for virtually any species, independent of genome size and ploidy. It is hence not surprising that TILLinG populations have been generated for various animal and plant species as described in (Kurowska et al., 2011).

While a range of methods have been developed for mutation detection in a TILLinG population (Yang et al., 2000; Colbert et al., 2001; Caldwell et al., 2004; Till et al., 2006; Raghavan et al., 2007; Suzuki et al., 2008; Dong et al., 2009), most were designed for the identification of mutations in a relatively small set of genes and become costly and labor-intensive when scaled to hundreds of genes. While pooling strategies (Tsai et al., 2011; Chi et al., 2014) combined with next-generation sequencing (NGS) have increased the number of genes (amplicons) that can be interrogated simultaneously, the background error rate can be high due to the numerous PCR steps in the protocol. In polyploid species, the presence of homeologs can additionally lead to false negatives and the interpretation of the sequence data may require customized bioinformatics pipelines (Tsai et al., 2011).

An alternative approach is to integrate NGS with capture methodologies. Saintenac et al. (2011) demonstrated that sequencing of DNA targeting non-repetitive genic regions can be highly reproducible and region-/locus-specific which can allow large-scale variant discovery in tetraploid wheat. Since sequence-capture methodologies offer the possibility of restricting sequencing to the coding portion of the genome, i.e., the exome (Winfield et al., 2012; Allen et al., 2013), they are especially suited to species with large or highly repetitive genomes, like wheat, where whole-genome sequencing would be excessively expensive (Tucker et al., 2011; Henry et al., 2014).

Exome capture probe design requires knowledge of the gene sequences preferably from full-genome assemblies. However, with a total of ∼16 Gbp for bread wheat and ∼11 Gbp for durum wheat, the wheat genome is one of the largest in the grass family, and full-genome assemblies of hexaploid and tetraploid wheat have only recently been released (IWGSC, 2018; Maccaferri et al., 2019). Therefore, all commercially available exome capture platforms were developed from wheat gene sequences in public databases such as NCBI and TriFL-DB (RIKEN) and EST and transcriptome assemblies. This carries the risk of underrepresenting low abundance genes and tightly regulated gene family members. Since exome capture is a hybridization process, not only will (near) identical sequences be captured but also non-target sequences (also called off-target reads) depending on the probes’ lengths and GC contents (Asan et al., 2011; Chilamakuri et al., 2014). Off-targets can include adjacent intronic regions, closely related genes, or homeologous sequences. Without an annotated reference sequence or knowledge of the complete gene set of an organism, these off-target sequences may be mistaken for allelic variants of a target gene. Thus, the potential for off-target alignments must be considered during the analysis and interpretation of mutant read alignments to mitigate false-positive mutation calls.

We developed a TILLinG population suitable for southern Australian environmental conditions. We chose an advanced spring-habit breeding line semi-dwarf tetraploid durum wheat (vernalization- and photoperiod-insensitive) which yields well in southern Australia and has given rise to the commercially grown cultivar DBA-Aurora. We used a subset of the population, 99 M_2_ plants, for an exome capture experiment using the Roche NimbleGen Wheat Exome Design. To overcome the complications caused by the Roche NimbleGen incomplete reference sequence for read alignment, such as missing homeologs, gaps, undefined nucleotides (i.e., “N”), and presence of homopolymer artifacts, we devised a novel method to construct a suitable reference sequence for mutation calling. We developed a bioinformatics pipeline for mutation calling and a web client application for querying and retrieval of mutation information.

## Materials and Methods

### Plant Material

Approximately 2,000 seeds from three individual plants of an advanced *Triticum turgidum* durum F2:5 breeding line (ex:UAD0951096 with the pedigree Tamaroi*2/Kalka//RH920318/Kalka///Kalka*2/Tamaroi) were mutagenized with 0.7% ethyl methanesulfonate (EMS) by gentle agitation in the solution on an orbital shaker overnight (18 hr) as described by Dong et al. (2009). Following rinsing, four seeds per pot were sown in 12-cm pots filled with coco peat with additional slow release fertilizer. After 20 days, when 76% of seeds had germinated, plants were thinned to one plant per pot in order to obtain a population of 500 mutant plants. Main spikes were isolated in bags pre-anthesis to ensure self-pollination. At full maturity, seeds were harvested separately from each mutant plant.

### DNA Isolation and Exome Capture

DNA Isolation and Exome Capture was extracted from a single 2-week old seedling of 99 randomly chosen M2 mutant plants and the unmutagenized control as described by Pallotta et al. (2000). Library preparation and hybridization followed Jordan et al. (2015) with modifications. Briefly, 1mg gDNA was fragmented by sonification to an average fragments length of 300bp. Illumina TruSeq libraries were prepared with fragmented DNA, indexed, size-selected, and pooled (n = 6) for exome capture. Pooled libraries were hybridized using the Roche’s NimbleGen wheat exome capture design (120426_Wheat_WEC_D02) () and protocol as described in Jordan et al. (2015).

### Building a Durum Exome Reference Sequence

Available genome sequences for the two tetraploid durum wheat cultivars Kronos (‘Kronos EI v1’) and Svevo (Maccaferri et al., 2019) were used for read alignment. In total, 245M paired-end reads of the unmutagenized control line UAD0951096_F2:5 were processed including adapter and quality trimming by Trimmomatic 0.36 (Bolger et al., 2014) using the following parameters: ILLUMINACLIP : TruSeq3-PE.fa:2:30:10 and LEADING:22 TRAILING:22 SLIDINGWINDOW:4:15 MINLEN:50.

The resulting reads were aligned using BioKanga version 4.3.6 (Stephen, 2012) (align –pemode 1 -s 2) to the Svevo pseudomolecules allowing for a 2% mismatch rate and no gaps. This resulted in 60% mapped reads. Since the genomic annotation for Svevo was not available when we conducted the project, we developed an in-house Java application (https://github.com/CroBiAd/TILLinG-mutants) for the retrieval of coding regions by making use of the coverage depth of aligned reads as an indicator. Start and end positions of genomic regions with a coverage of 17 reads or more were firstly marked and subsequently retrieved together with 500-bp flanking sequences on either side. The reason to add these tails was the observation that coverage never drops abruptly at the intron–exon boundaries of exome captured aligned reads. If two regions with high coverage were in close proximity, i.e., less than 301 bp, they were merged (**Supplementary Figure 1**). The resulting 191,892 contigs covered a total length of 443 Mbp.

In the second step, reads that did not map to the Svevo genome (97 M) were aligned to Kronos by BioKanga as above resulting in 10.6% mapped reads. Regions were retrieved as described above. In the third step, the remaining 86.7 million unaligned reads were assembled using ABySS version 2.0.2 (Jackman et al., 2017) with k-mer size = 96. We selected contigs with a minimum length of 500 bp resulting in 552 contigs with a total length of 420 Kbp. We performed BlastX searches to explore which proteins might potentially be encoded by the 552 assembled contigs against rice (MSU Rice Genome Annotation Project Release 7) (Kawahara et al., 2013) and *Arabidopsis* (TAIR10) (Berardini et al., 2015) protein sequence databases (e-value cutoff 10^−5^).

Combining the three sets of contigs (i.e., from Svevo, Kronos, and the ABySS assembly) gave us our 484.4 Mbp reference sequence for read alignment and mutation detection, hereafter called DECaR (DurumExomeCaptureReference). DECaR can be downloaded from doi: 10.25909/5d258fa699358

### Read Alignment to Decar and Mutation Calling Pipeline

Following quality and adapter trimming, exome captured reads (minimum 50 bp) from unmutagenized control line, and the M2 lines were aligned to DECaR using Bowtie 2 version 2.3.0 (Langmead and Salzberg, 2012) allowing a 2% mismatch rate with the following parameters: –end-to-end –very-sensitive –n-ceil L,0,0.1 –rdg 3,3 –rfg 3,3 –no-unal –mp 6,6 –np 4 –no-mixed -score-min L,0,-0.12

After alignment PCR duplicates were detected and removed from BAM files using our in-house Java application.

One pileup file was generated from the bam files using SAMtools version 1.6 (Li et al., 2009) with a minimum mapping quality (MAPQ) of 2 to mitigate multi-mapping and mapping errors.

We used three criteria to identify mutations in the TILLinG population. First, any variation from DECaR was considered a potential mutation if it was present in only one mutant sample and non-polymorphic between the control line and DECaR. Secondly, we demanded a mutation to be covered by at least three reads to be confident that the mutation was not derived from sequencing error. Finally, because coverage at a reference position was variable from sample to sample, a mutation was only called in a mutant sample if we had sufficient coverage for the control allele in at least 50 other mutant samples. An in-house developed Java application was used to implement this logic

Initially, 9.5M mutations were called across all the 81 mutant samples that had sufficient coverage. In order to reduce false-positive calls, we applied two conditions that had been used in previous studies (Henry et al., 2014; King et al., 2015). Firstly, EMS preferentially changes C- > T and G- > A. It has been shown that the higher percentage of CG- > TA transitions in EMS-induced mutant populations was associated with better mutation calling (Henry et al., 2014; King et al., 2015). Secondly, we expected a ratio of 2:1 heterozygous to homozygous mutations in M_2_ populations (Henry et al., 2014).

### Database

To make the results easily accessible, we created a Web application for querying the mutations.

First, details of all detected mutations (i.e., position, zygosity, flanking sequence) were deposited into an SQLite database. Then, the stand-alone version of BLAST^©^ Command Line Application (ncbi-blast-2.7.1+) (Camacho et al., 2009) was installed locally, and a nucleotide BLAST database was generated from the DECaR. Next, an ASP.Net 4.6 Web client, published on Microsoft Internet Information Services (IIS), was developed to allow BLAST searches of the DECaR and querying of the mutations. Finally, the complete application, named Durum Wheat TILLinG (DuWTill) was hosted on Microsoft Windows Server^©^ 2016 Standard edition and is publicly accessible at http://duwtill.acpfg.com.au/.

In addition, DuWTill is available for download through GitHub (https://github.com/CroBiAd/DuWTill) where steps to build it locally are described. After installation DuWTill can be run locally either with our data or on data sets prepared by researchers from their own populations. The distribution is provided for the Windows operating system, which requires Windows IIS to be turned on and Visual Studio 2015 or later () installed on the development computer.

## Results

### The Novel Durum Exome Reference Sequence DECaR

We mapped reads from the unmutagenized control line to the two publically available durum wheat genome assemblies [Svevo (Maccaferri et al., 2019) and Kronos (‘Kronos EI v1’)]. By combining these aligned read data with contigs assembled from unmapped reads, we constructed a new durum exome reference, DECaR, which consists of 220,114 contigs with a total length of 484,479,862 bp covering ca. 4% of the estimated 11-Gbp durum wheat genome. A comparison of alignment rates of the control sample to the NimbleGen reference and DECaR showed 20% alignment *versus* 51%, respectively. We also observed an increase in average alignment quality (MAPping Quality, MAPQ, SAMtools (Li et al., 2009)) from 28.75 to 29.74.

**Figure 1:**
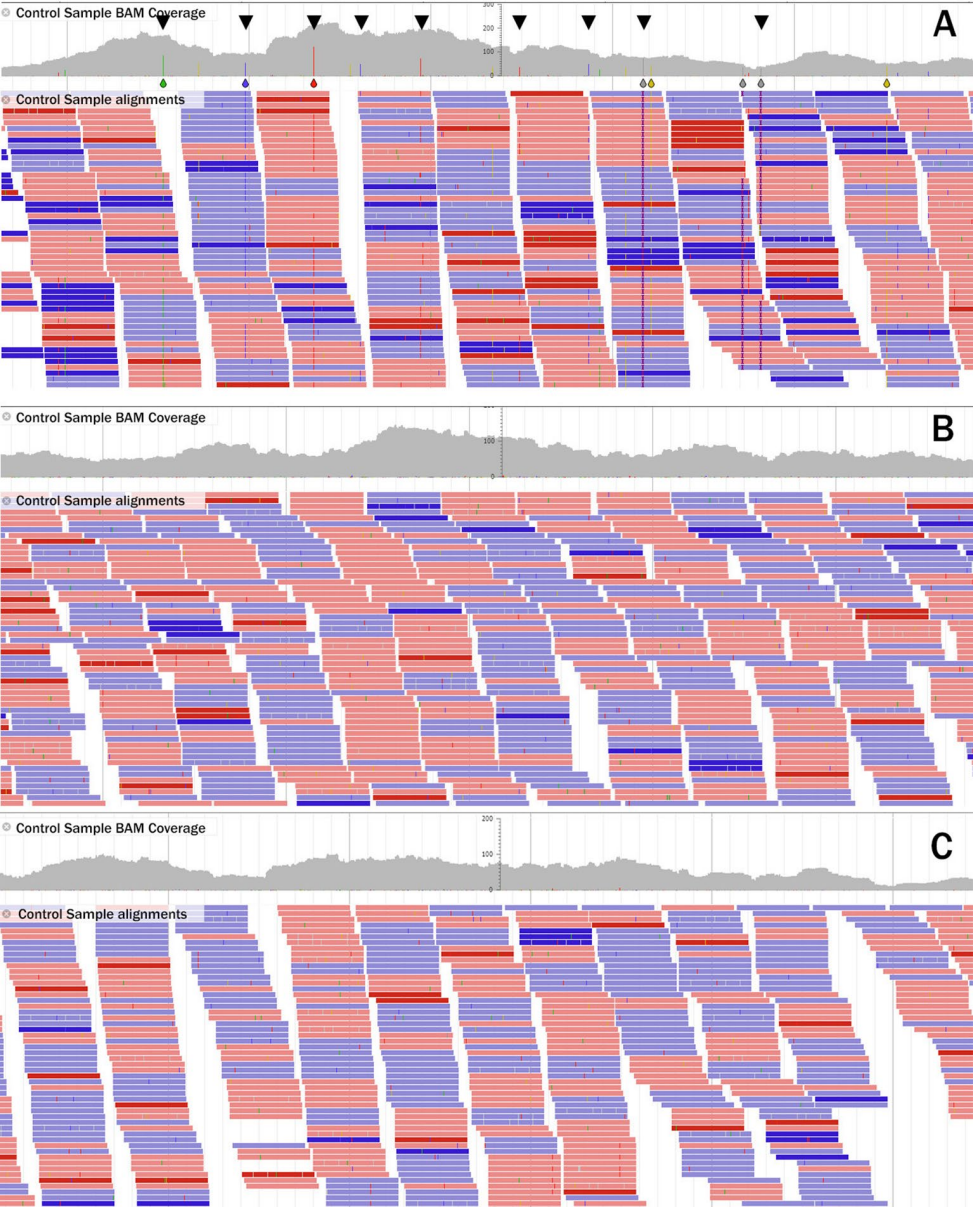
Example of read alignment of the control line to the a 1.37-Kbp region of **(A)** the Roche NimbleGen exome reference contig05736, **(B)** to the corresponding regions of contig05736 in DECaR originating from Svevo chromosome 3A, and **(C)** Svevo chromosome 3B. Reads in red align to the (+) strand, those in blue to the (-) strand. Location of potential mutations/polymorphisms are indicated by blue (cytosine), green (adenine), red (thymine), and yellow (guanine) bars for the DNA base called and highlighted by black triangles in the coverage track in A; in B and C, no mutated bases were called.

JBrowse was used to visually compare the alignment of the unmutagenized control sample reads to the original Roche NimbleGen exome reference and to DECaR, respectively. **Figure 1A** shows reads aligned to contig05736 of the original Roche exome reference. In this example, it is clear that, within the 1.37-kbp region, there are several putatively mutated/polymorphic positions (depicted as colored bars in the coverage track and indicated by black triangles). **Figures 1B, C** show alignments of the same reads to the corresponding regions located on DECaR contigs derived from chromosomes 3A and 3B, respectively. No mutated positions are visible. This example demonstrated the advantage of DECaR to position reads properly to the A and B chromosomes, whereas alignments of the same reads to the NimbleGen exome capture reference created false positives.

### Mutations’ Discovery

The average read coverage per base position in all samples was estimated, and results are given in **Supplementary Table 1**. Coverage within mutant samples ranged from 1.2 to 11.3 with an average of 6 (reads/base position). Alignment rates ranged from 13 to 76% with an average of 57%. In order to find the reason for a low alignment rate for some samples, we selected the three samples with the lowest alignment percentage (673, 677, 661) and mapped their reads to the entire Svevo reference genome. Surprisingly, alignment rates increased to 77, 81, and 80% respectively. Closer inspection indicated that these samples contained a significant amount of non-exonic DNA; however, they nevertheless showed sufficient coverage in the coding regions to be included. On the other hand, we excluded samples with low coverage either due to lower exome capture efficiency or high PCR duplication rates. In summary, for 18 of the 99 mutant samples, the data failed to be of sufficient quality to proceed; therefore, these were excluded from further analysis (**Supplementary Table 1**).

**Figure 2:**
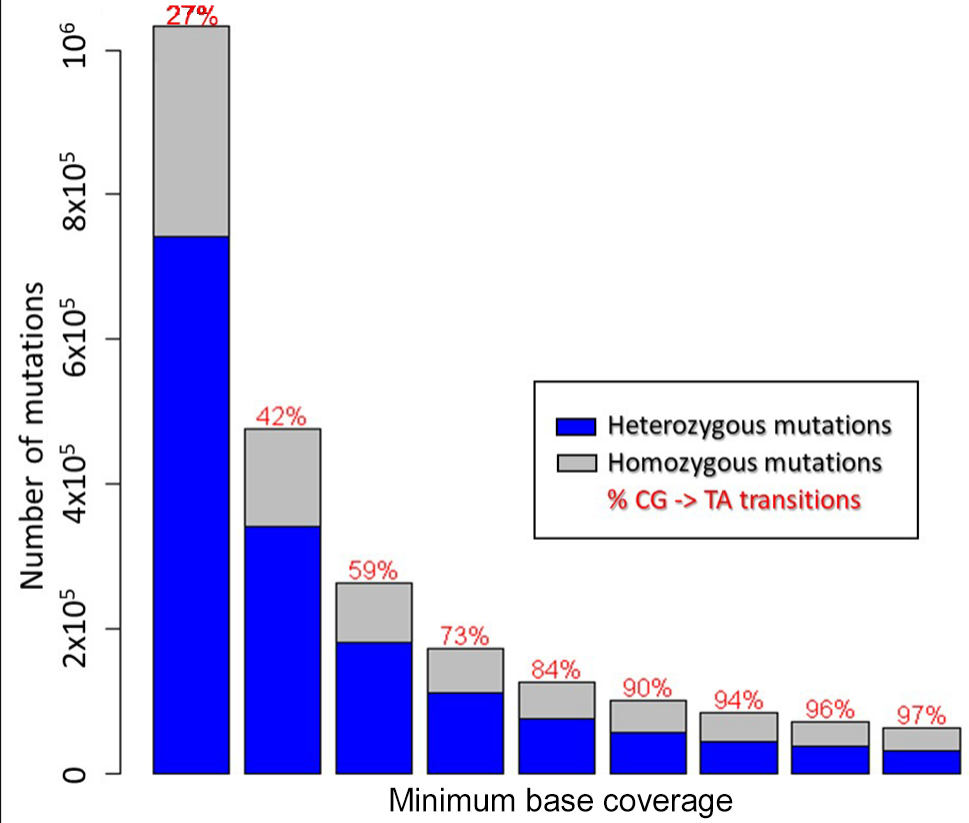
Number of mutations and their zygosity depending on mutant allele coverage.

To reduce false-positive calling of mutations, we gradually increased the minimum number of reads confirming a mutated base as shown in **Figure 2**. **Table 1** shows the results for a minimum coverage of 10, for which 83,573 mutations were called (49,652 heterozygous, 33,921 homozygous) of which 94% were of CG- > TA type.

**Table 1:** Types of mutations and their frequencies detected in the 81 mutant lines for a minimum base coverage of 10.

Mutation	Base	Number
Deletion	A	19
Deletion	C	15
Deletion	G	23
Deletion	T	20
Substitution	A- > C	53
Substitution	A- > G	285
Substitution	A- > T	277
Substitution	C- > A	1,705
Substitution	C- > G	51
Substitution	C- > T	39,294
Substitution	G- > A	39,472
Substitution	G- > C	34
Substitution	G- > T	1,610
Substitution	T- > A	385
Substitution	T- > C	240
Substitution	T- > G	90
SUM		83,573

Since coverage varied from sample to sample, the number of detected mutations per sample ranged from 12 (in mutant sample 653) to 2,603 (in mutant sample 417) (see **Supplementary Table 1**). The mutation rate among the 81 samples varied from 2.4 to 20.7 mutations/Mbp, with an average of 10.3 (derived by dividing the total number of mutations by number of positions that are covered by 10 reads or more).

The unmutagenized control sample was sequenced to a higher depth, and average base coverage (29.5 reads/base position) was deeper (>3 times) than that of mutant samples (**Supplementary Table 1**). The higher coverage of the control sample helped us to distinguish SNPs specific to the line from true EMS-derived mutations.

Svevo is an Italian durum wheat cultivar derived from crossing CIMMYT selection with Zenit in the 1990s. Kronos, on the other hand, was released by Arizona Plant Breeders in 1992 and is derived from a male-sterile-facilitated recurrent selection population (APB MSFRS Pop, selection D03–21) (Jackson, 2011; Berg, 2014). Whereas the advanced breeding line used in our study has Australian cultivars Kalka adapted to the boron-toxic soil of Southern Australia and Tamoroi in its pedigree. A recent study into genetic diversity across durum wheat by Kabbaj et al., 2017 shows that the Australian cultivars are distinct from Kronos and Svevo. It is therefore not surprising that we not only saw varietal SNPs but also differences in gene content between the accessions. One example of gene families that rapidly evolved is the NBS-LRR disease resistance genes (Steuernagel et al., 2016). These tend to vary significantly between elite cultivars due to selective breeding. Indeed, we found that 41 of the assembled contigs showed homology to disease resistance genes, but there were also members of the cytochrome P450 and oxidoreductase families (**Supplementary Table 2**).

### The DuWTill Database

We deposited all identified mutations into a database and developed the online tool DuWTill for access to the collection. DuWTill is publicly available at http://duwtill.acpfg.com.au/.

**Figure 3:**
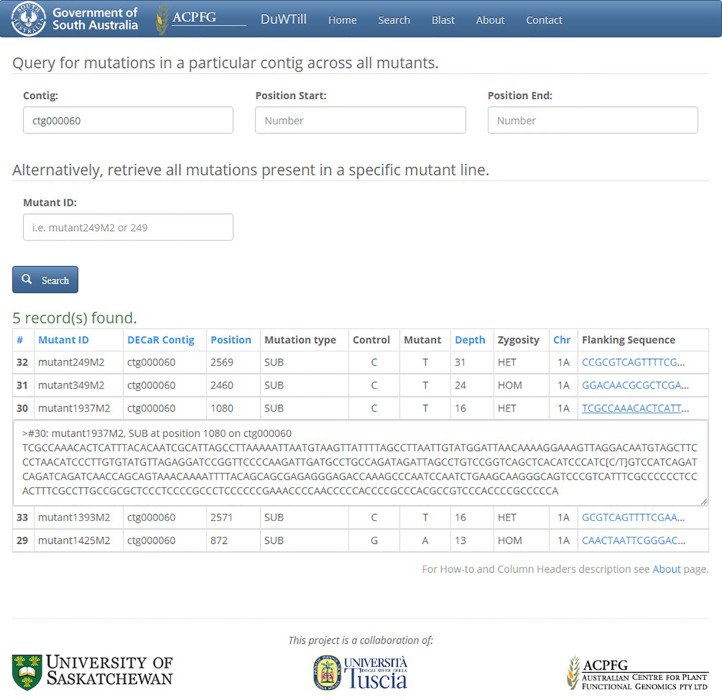
Search page showing results of query in DuWTill by contig ID. Output table, sorted by depth, displays all mutations within DECaR contig ctg000060 detected across the mutants. Third row (mutant1937M2) is expanded to show the full flanking sequence in FASTA format.

DuWTill’s intuitive interface has principally one main ‘Search’ page (**Figure 3**) where the mutations table is displayed. The database can be searched by two types of identifiers (with restriction on region, if preferred):

1. Contig ID (using DECaR nomenclature) to obtain mutations for all mutant lines occurring within a specific contig.
2. Mutant ID (individual mutant plants) to get mutations on all contigs for a specific mutant line.

The output table contains one row for each mutation found and the mutation position (in bases) relative to the start of the respective DECaR contig, induced mutation type, base call in the non-mutagenized control and in the EMS-mutagenized individual. It also includes predicted zygosity, chromosome location, and mutant allele coverage as a measure of confidence that the mutation has been called correctly. Clicking on the flanking sequence link will expand the sequence fragment with minimum 50 to 200 bp on either side of the putative mutation.

**Figure 4:**
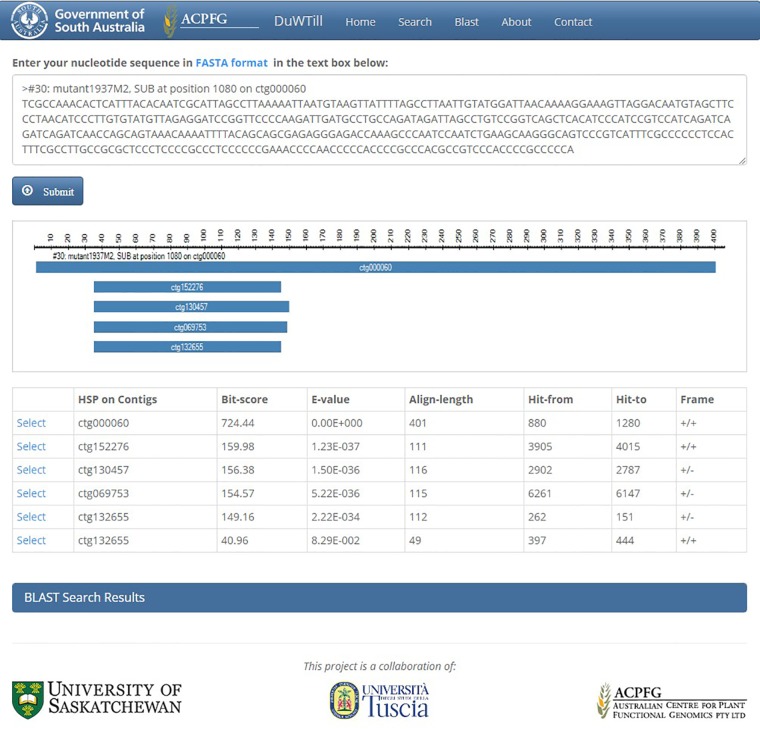
BLAST page showing the results of a BLASTn search query in DuWTill. BLASTn search was executed using the same sequence that was presented in the table of **Figure 3**. Top hits to DECaR are shown in two views: alignment overview and hit table. Detailed HSP alignments are reported in the additional expandable panel “BLAST Search Results.”

Alternatively, the DECaR can be queried with a FASTA-formatted sequence of interest using the internal BLAST portal on a separate utility “BLAST” page (**Figure 4**). The top hits to the available reference will be displayed. Selecting a hit will redirect to the “Search” page showing all putative mutations in the sequence of interest called within a contig for all mutants of the population.

In addition, short background information, how-to instructions and a description of the table headers are presented on the “About” page.

DuWTill source code and mutation call table were deposited in a public repository on GitHub (https://github.com/CroBiAd/DuWTill). It also includes a console application, YourDB, which has been written to help with formatting the mutation calls into an SQLite database.

The DuWTill application can be used for TILLinG data from any organism and is also independent of the mutation calling pipelines. YourDB application will format any comma separated value (.csv) file which contains the appropriate fields into an SQLite database. Obviously, if the TILLinG data does not make use of DECaR, the exome reference BLAST database needs to be substituted with an appropriate one. The GitHub repository contains all necessary instructions.

## Discussion

We tested the suitability of the Roche NimbleGen wheat exome capture platform for mutation discovery in a subset of M2 plants generated with EMS of an Australian adapted breeding line. As researchers before us had, we faced the challenge of developing a mutation calling pipeline that would maximize true positive calls and minimize false positives but not result in missing many potentially interesting mutations. For example, Henry et al. (2014) applied their bioinformatics pipeline (MAPS) which was initially designed for detection of mutations in EMS-mutagenized rice to exome capture data of the Kronos and six tetraploid M2 wheat plants derived from Kronos. The researchers used the Roche NimbleGen exome capture reference contigs for alignment of the captured reads using the short-read aligner BWA-SW. A variant was called a mutation if it was present in only one of the samples and absent in Kronos, based on the assumption that the probability of the same mutation appearing in two independent plants is low. The applied minimum read coverage was set to seven for heterozygous and five for homozygous mutations and resulted in more than 90% of CG- > TA mutation rate. Setting the minimum coverage threshold to a higher value will decrease the false-positive rate but can simultaneously also reduce the number of true mutations called. In other words, the mutations with lower coverage are not all false. For our dataset and using the DECaR reference, we chose a minimum coverage of at least 10 reads per base position based on 94% of mutations called being CG- > TA transitions. By settling on a minimum coverage of 10 reads per base position for a mutation call, we erred on the side of caution in order to keep false positives low. This may explain the comparatively lower average mutation rate of 10.3/Mbp and a heterozygous to homozygous ratio of 1.47. The stringency of mutation calling can be adjusted by for example choosing a lower read depth/base position and a different minor allele ratio when running our Java script. Thus, researchers have the flexibility to analyze the data to what is most appropriate for their needs.

Wheat exome capture designs have developed along with improved sequence and genome assembly knowledge. King et al. (2015) used a custom designed capture array with 1,846 full-length cDNAs (approximately 2 Mbp capture space) for targeted sequencing to examine *TaGA20ox1* homeologs across three Cadenza bread wheat EMS-induced M5 mutant lines. The sequence reads were aligned to the IWGSC Chinese Spring–derived Chromosome Survey Sequences CSS (IWGSC, 2014). They obtained a relatively low alignment rate of 26% to target genes compared to similar targeted capture experiments. Despite low alignment rates, by filtering based on CG- > TA mutation rates and expected hetero-homozygous ratios, King et al. (2015) were able to validate 75–80% mutations called.

Another significant wheat EMS-induced exome capture experiment was performed by Krasileva et al. (2017). In that study, first, a new and improved whole-exome capture design was developed that targeted 84 Mbp sequence space. One thousand and three hundred thirty-five EMS-induced M_2_ mutants of the tetraploid cultivar Kronos and 1,200 mutants of the hexaploid, bread wheat cultivar Cadenza were exome captured and sequenced. One hundred base pairs of paired-end reads were aligned to A and B genome contigs of the CSS (IWGSC, 2014). Similarly to our study, Krasileva et al. (2017) then improved their reference in order to improve alignment. They did this by assembling the remaining unmapped reads from their samples to expand their sequence-capture space by an additional 33.4 Mbp for the durum, i.e., 117.4 Mbp in total.

With the availability of two full durum wheat reference sequences and the recently released assembly (Maccaferri et al., 2019), we were able to create a new reference specifically for this durum wheat exome capture experiment. By combining regions from the Svevo and Kronos genomes (where reads from the control line aligned) with contigs assembled from unaligned reads, we created a 484.4 Mbp new reference which was more than four times larger than the original Roche reference (106.9 Mbp) (). The process of first aligning reads from our deeper-sequenced unmutagenized control sample also allowed us to distinguish varietal SNPs confidently. This approach could be used for any new TILLinG population where knowledge of the complete gene set is not known.

King et al. (2015) also demonstrated that absence of one or two copies of a gene in the wheat reference could cause homozygous mutations to be erroneously called heterozygous, because reads containing the mutated position were diluted by wild-type reads. A true mutation located in a homeolog that is not represented in the reference sequences can lead to its assignment to the wrong homeolog (off-target homeolog). Roche’s NimbleGen Exome capture reference is mostly homeolog-insensitive and was designed by including sequences from multiple hexaploid wheat varieties generated by different sequencing technologies. The advantage of using DECaR over the NimbleGen reference is: having a durum-based reference, including absent homeologs, an improved reference sequence quality (e.g., removal of homopolymer errors, and inclusion of intronic regions for better mapping); to be as inclusive as possible (i.e., include genes that were captured but not represented in the original reference); and finally to keep the alignment space small for ease of computation. Following this adjustment, the mutation rate was estimated to be 20.1 mutations/Mbp, consistent with a previously reported mutation rate (Uauy et al., 2009).

The DuWTill database was developed as a tool to mine for mutants of interest following exome capture. We required an intuitive interface for collaborators to query the data and obtain sufficient information for follow-up work such as primer design to test for the presence of the mutation of interest. Until very recently, no such tools existed and especially not for durum wheat.

DuWTill application was designed to accommodate information on a large number of individuals and their mutations and is easily adaptable to other organisms than durum wheat. It is a small and simple tool which can be easily installed locally on any windows platform even a laptop, or can be run as an open web service application.

For wheat, DuWTill is comparable with the established and widely used database at wheat-tilling.com which houses mutants’ and mutations’ information from Krasileva et al. (2017). The wheat-tilling database additionally incorporates useful mutation effects and oligo primer designs where these have been predicted or tested, whereas DuWTill does not. The DuWtill interface has been designed to be simple, portable, and user-friendly and displays flanking sequence with the mutation in position for primer design on the same page as all other information. However, we believe its main advantage is the ability to readily update the reference which should continue to make it an effective tool for mining variant information for the future.

## Conclusions

We have optimized a reference sequence for tetraploid wheat to use with the Roche Wheat Exome Capture Design for diversity and mutation studies. Furthermore, we have developed a bioinformatics pipeline for the analysis of TILLinG mutants in conjunction with the new reference and have called mutations for a subset of an Australian durum TILLinG population. A software application has been written that allows online or local interrogation of the TILLinG collection and can also be used to host propriety data.

All resources are publically available to interested researchers and can be adapted to their needs.

## Data Availability Statement

The sequence data generated for this study can be found in SRA under https://dataview.ncbi.nlm.nih.gov/object/PRJNA574238?reviewer=lbstjt0r17312aeo6n7nqjdtv6

## Author Contributions

MF analysed the exome capture data and wrote the in house Java application. EK designed and programmed the DuWTill. PK isolated the DNA; JE and KW made the libraries and performed exome capture experiments. UB, MF, EK, PT, and CP wrote the manuscript; CP, PT, and UB designed the study.

## Funding

This project was supported by a Premier’s Research and Industry Fund grant (no. IRGP15) provided by the Government of South Australia Department of State Development.

## Conflict of Interest

The authors declare that the research was conducted in the absence of any commercial or financial relationships that could be construed as a potential conflict of interest.

## Supplementary Material

Supplementary Table 1 Detailed information on alignment rates and mutations for each Tilling line.

Supplementary Table 2 BLAST search results for the assembled contigs.
