# Supplementary material for: Novel Informatic Tools to Support Functional Annotation of the Durum Wheat Genome
Source: Front Plant Sci. 2019 Oct 10;10:1244. doi: 10.3389/fpls.2019.01244 (PMC6795695; doi:10.3389/fpls.2019.01244)
Supplement: Supplementary Table 1 — Detailed information on alignment rates and mutations for each Tilling line. [file Table_1.docx]

**
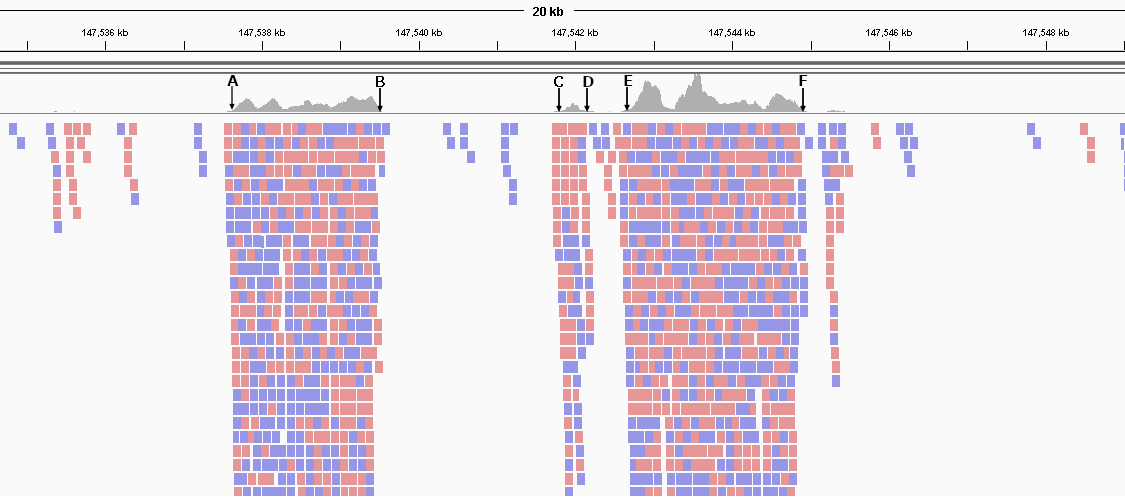
**

**Supplementary Figure 1:** Visualisation of read alignment in Integrated Genome Viewer.

The start and end point of regions with high read depth were first marked (AB, CD and EF) and subsequently extracted together with 500 bp flanking sequence from the Svevo (or Kronos genome). Regions CD and EF that are close to each other were merged into one region. Reads aligning to the DNA (+) strand are in red, whereas those aligning to the (-) strand are in blue.

**Supplementary Table 1**

| **Sample number** | **Mapping Rate** | **PCR Duplication Rate** | **Coverage**  **Per base** | **Mutations** | **Position with Coverage ≥10 (in Millions)** | **Mutation Rate** |
| --- | --- | --- | --- | --- | --- | --- |
| Control | 51% | 0.27 | 29.5 |  | 227 |  |
| 1313 | 51% | 0.33 | 8.2 | 1130 | 122 | 9.2 |
| 1321 | 55% | 0.31 | 9.5 | 1385 | 132 | 10.5 |
| 1341 | 50.31% | 0.64 | 3.1 |  | 45 |  |
| 1349 | 66.14% | 0.30 | 8.4 | 2000 | 127 | 15.8 |
| 1353 | 54% | 0.29 | 10.5 | 1173 | 139 | 8.5 |
| 1373 | 35.82% | 0.44 | 3 | 257 | 40 | 6.4 |
| 1381 | 48.38% | 0.63 | 3 |  | 44 |  |
| 1389 | 65.10% | 0.30 | 8.4 | 1075 | 124 | 8.7 |
| 1393 | 70.30% | 0.32 | 8.7 | 1968 | 133 | 14.8 |
| 1409 | 34.20% | 0.44 | 3 | 403 | 39 | 10.4 |
| 1417 | 67.53% | 0.30 | 9.3 | 1724 | 136 | 12.7 |
| 1425 | 69.69% | 0.39 | 6.3 | 1035 | 92 | 11.3 |
| 1433 | 71.47% | 0.39 | 7.5 | 1369 | 105 | 13.0 |
| 1441 | 47.50% | 0.63 | 4.2 |  | 56 |  |
| 1461 | 73.38% | 0.39 | 8.4 | 1799 | 113 | 15.9 |
| 1469 | 70.93% | 0.39 | 7.6 | 504 | 106 | 4.8 |
| 1693 | 73.69% | 0.40 | 9.4 | 1241 | 117 | 10.6 |
| 1733 | 73.72% | 0.39 | 9.9 | 1423 | 121 | 11.8 |
| 1741 | 32.18% | 0.44 | 2.8 | 232 | 35 | 6.6 |
| 1897 | 70.68% | 0.42 | 11.3 | 2313 | 137 | 16.9 |
| 1901 | 65.97% | 0.40 | 5 | 582 | 79 | 7.4 |
| 1905 | 64.62% | 0.41 | 6.5 | 986 | 94 | 10.5 |
| 1909 | 40.81% | 0.42 | 3.3 | 255 | 46 | 5.6 |
| 1913 | 70% | 0.32 | 8.4 | 1512 | 130 | 11.6 |
| 1929 | 42.12% | 0.43 | 4.8 | 1013 | 65 | 15.6 |
| 1933 | 40.40% | 0.41 | 3.2 | 366 | 42 | 8.7 |
| 1937 | 70.54% | 0.42 | 6.9 | 1249 | 101 | 12.3 |
| 1941 | 56.12% | 0.28 | 8.6 | 1396 | 131 | 10.7 |
| 1961 | 55% | 0.29 | 7.5 | 974 | 117 | 8.3 |
| 1969 | 57.56% | 0.28 | 8.7 | 1803 | 131 | 13.7 |
| 1973 | 51.70% | 0.65 | 3.6 |  | 51 |  |
| 1977 | 69.36% | 0.41 | 9.4 | 1511 | 124 | 12.2 |
| 1985 | 39.12% | 0.42 | 3.9 | 666 | 51 | 13.1 |
| 1997 | 40.33% | 0.44 | 3.7 | 506 | 51 | 9.9 |
| 245 | 71.74% | 0.25 | 7.5 | 862 | 105 | 8.2 |
| 249 | 72.14% | 0.27 | 8.4 | 1029 | 113 | 9.1 |
| 285 | 39.46% | 0.45 | 2.8 | 257 | 37 | 6.9 |
| 289 | 76.05% | 0.26 | 9.5 | 1844 | 127 | 14.6 |
| 293 | 72.05% | 0.23 | 8.6 | 1456 | 115 | 12.7 |
| 309 | 70.22% | 0.24 | 5.8 | 891 | 87 | 10.2 |
| 317 | 36.86% | 0.45 | 3 | 385 | 39 | 9.8 |
| 321 | 38.24% | 0.45 | 3 | 291 | 42 | 7.0 |
| 329 | 71.84% | 0.28 | 8 | 1458 | 117 | 12.5 |
| 333 | 73.49% | 0.31 | 5.8 | 849 | 93 | 9.1 |
| 349 | 74.60% | 0.28 | 6 | 741 | 97 | 7.6 |
| 353 | 69.51% | 0.23 | 10 | 1175 | 120 | 9.8 |
| 357 | 68.48% | 0.32 | 10 | 1729 | 130 | 13.3 |
| 369 | 72.93% | 0.32 | 8 | 831 | 108 | 7.7 |
| 373 | 71.10% | 0.25 | 7.6 | 685 | 106 | 6.4 |
| 381 | 73.01% | 0.29 | 7.8 | 1071 | 112 | 9.6 |
| 389 | 63.28% | 0.32 | 6.6 | 995 | 94 | 10.6 |
| 393 | 66.01% | 0.34 | 4.4 | 632 | 70 | 9.1 |
| 397 | 65.53% | 0.32 | 6.2 | 872 | 93 | 9.4 |
| 405 | 74.30% | 0.29 | 8 | 1070 | 118 | 9.1 |
| 409 | 68.96% | 0.33 | 5.4 | 376 | 85 | 4.4 |
| 413 | 65.39% | 0.35 | 6 | 839 | 88 | 9.5 |
| 417 | 67.40% | 0.35 | 10.5 | 2603 | 126 | 20.7 |
| 421 | 62.57% | 0.35 | 7 | 1164 | 98 | 11.9 |
| 425 | 69.19% | 0.33 | 6.4 | 1340 | 95 | 14.1 |
| 429 | 69.23% | 0.34 | 8.3 | 1426 | 112 | 12.7 |
| 441 | 68.57% | 0.34 | 6 | 798 | 88 | 9.1 |
| 445 | 70.24% | 0.33 | 8.2 | 1705 | 110 | 15.5 |
| 449 | 65.10% | 0.36 | 7 | 1087 | 96 | 11.4 |
| 453 | 68.44% | 0.31 | 9.3 | 1678 | 123 | 13.6 |
| 457 | 16.65% | 0.27 | 2 | 70 | 14 | 5.1 |
| 465 | 64.30% | 0.35 | 7.2 | 1486 | 101 | 14.7 |
| 469 | 50.04% | 0.66 | 1.3 |  | 21 |  |
| 477 | 58.80% | 0.35 | 7 | 1092 | 98 | 11.2 |
| 489 | 16.34% | 0.28 | 1.4 | 17 | 7 | 2.4 |
| 505 | 33.01% | 0.58 | 2.4 |  | 32 |  |
| 509 | 37.42% | 0.59 | 2.5 |  | 34 |  |
| 529 | 34.22% | 0.58 | 2.7 |  | 36 |  |
| 533 | 72.06% | 0.41 | 7 | 1231 | 105 | 11.7 |
| 573 | 55.10% | 0.46 | 5.2 |  | 83 |  |
| 581 | 56.68% | 0.46 | 6.3 |  | 96 |  |
| 585 | 52.91% | 0.45 | 5.8 |  | 90 |  |
| 589 | 52.48% | 0.45 | 5.3 |  | 82 |  |
| 593 | 55.10% | 0.46 | 6 |  | 93 |  |
| 641 | 57.14% | 0.46 | 5.5 |  | 86 |  |
| 653 | 15.40% | 0.27 | 1.2 | 12 | 5 | 2.5 |
| 661 | 16.06% | 0.27 | 1.7 | 45 | 9 | 4.8 |
| 673 | 13.19% | 0.32 | 1.3 | 60 | 8 | 7.4 |
| 677 | 15.64% | 0.30 | 1.3 | 26 | 7 | 3.8 |
| 741 | 39.98% | 0.42 | 4 | 333 | 52 | 6.4 |
| 745 | 37.33% | 0.58 | 3 |  | 41 |  |
| 749 | 36.89% | 0.57 | 2.7 |  | 36 |  |
| 773 | 58.25% | 0.37 | 10 | 1950 | 121 | 16.2 |
| 777 | 51.69% | 0.28 | 7 | 1006 | 112 | 9.0 |
| 789 | 31.26% | 0.57 | 2.3 |  | 29 |  |
| 821 | 69.76% | 0.43 | 8 | 1719 | 111 | 15.6 |
| 825 | 72.52% | 0.43 | 5.5 | 856 | 86 | 10.0 |
| 849 | 56.64% | 0.29 | 6.7 | 989 | 105 | 9.4 |
| 853 | 69.00% | 0.41 | 7.5 | 984 | 104 | 9.4 |
| 857 | 71.38% | 0.41 | 8.4 | 960 | 116 | 8.3 |
| 861 | 70.79% | 0.41 | 8 | 1161 | 112 | 10.4 |
| 865 | 70.07% | 0.42 | 6 | 928 | 93 | 10.0 |
| 869 | 53.21% | 0.66 | 3.5 |  | 51 |  |
| 881 | 62.88% | 0.30 | 7.8 | 1518 | 120 | 12.6 |
| 885 | 53.60% | 0.28 | 6 | 1141 | 94 | 12.1 |
